# Circadian Alignment of Feeding Regulates Lifespan Extension by Caloric Restriction

**DOI:** 10.1093/geroni/igab046.442

**Published:** 2021-12-17

**Authors:** Victoria Acosta-Rodriguez, Filipa Rijo-Ferreira, Mariko Izumo, Pin Xu, Carla Green, Joseph Takahashi

**Affiliations:** 1 UT Southwestern Medical Center, Dallas, Texas, United States; 2 Icahn School of Medicine at Mount Sinai, New York, New York, United States

## Abstract

Caloric restriction (CR) promotes longevity in several species. Classic CR protocols often lead to chronic cycles of 2h-feeding/22h-fasting, raising the question whether calories, fasting or time of day are causal. To address this, we tested an AL control group and five CR protocols with different timing and duration of feeding/fasting cycles. C57BL/6J male mice were subjected to 30% CR as one single meal a day at the beginning of the day or night (classical protocols with < 2h feeding, CR-day and CR-night), or smaller meals distributed for 12h (CR-day-12h and CR-night-12h), or evenly spread out throughout 24h (CR-spread) to abolish the otherwise daily feeding pattern adopted by nocturnal animals. We found that CR alone is sufficient to extend lifespan without fasting. However, the benefits are enhanced if feeding/fasting cycles are present and match their normal nocturnal activity. Circadian alignment of feeding with at least 12h fasting boosts CR-mediated increase on survival in mice, independently body weight. Aging leads to widespread upregulation of inflammation-related genes and downregulation of metabolic pathways in liver from ad lib fed mice; whereas CR at night ameliorates these aging-related changes and preserves circadian oscillations in gene expression. Overall, our results demonstrate that circadian interventions promote longevity and provide a novel perspective for elucidating mechanisms of aging.

